# Hypoxia-relieving and glycolysis-disrupting polydopamine nanomedicine for synergistic chemo-photothermal therapy of hepatocellular carcinoma

**DOI:** 10.1016/j.ijpx.2026.100506

**Published:** 2026-02-18

**Authors:** Xiang Wang, Yihan Ma, Le Wang, Hengrui Li, Miao Qin, Ruonan Sun, Jing Hu

**Affiliations:** a Wuxi School of Medicine, Jiangnan University, Wuxi 214122, China; b School of Biotechnology and Key Laboratory of Carbohydrate Chemistry and Biotechnology of Ministry of Education, Jiangnan University, Wuxi 214122, China; c School of Food Science and Technology, Jiangnan University, Wuxi 214122, China; d Institute of Future Food Technology, JITRI, No. 19 Wenzhuang Road, Yixing 214200, China

**Keywords:** Hepatocellular carcinoma, Shikonin, Glycolysis, Pyruvate kinase M2, Chemo-photothermal therapy

## Abstract

Hypoxia is a hallmark of solid tumors that compromises therapeutic efficacy in hepatocellular carcinoma (HCC). Here, a multifunctional polydopamine (PDA)-based nanomedicine co-loading shikonin (SK) and catalase (CAT) and functionalized with galactose (Gal) is developed and termed SC@PDA-Gal. SC@PDA-Gal delivers SK to inhibit pyruvate kinase M2 (PKM2) and disrupt glycolytic output, while the co-delivered CAT decomposes endogenous H_2_O_2_ to generate O_2_ in situ, thereby downregulating HIF-1α and alleviating hypoxia. Under hypoxic conditions, SC@PDA-Gal reduces lactate production by 51% (vs. control) and depletes intracellular adenosine triphosphate (ATP) by 89% (vs. control) in HCC cells, indicating effective glycolysis suppression. Moreover, PDA enables efficient photothermal conversion under near-infrared (NIR) irradiation, providing localized hyperthermia and accelerating drug release. In vivo, SC@PDA-Gal achieves a tumor inhibition rate of 93.44 ± 2.86% in subcutaneous C5WN1-bearing mice with favorable biosafety. Collectively, SC@PDA-Gal represents a targeted, hypoxia-adaptive chemo-photothermal nanomedicine for precision HCC therapy.

## Graphical abstract

**Figure:**
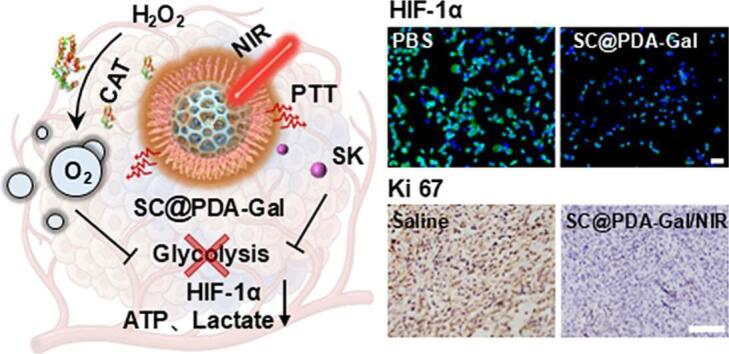

## 1 Introduction

Hepatocellular carcinoma (HCC) is a major global health challenge, representing the majority of primary liver cancer cases and ranking third in cancer mortality (Lee et al., 2022; Li et al., 2024b; Wen et al., 2022). A defining feature of HCC is hypoxia, which acts as a critical driver of drug resistance and tumor progression (Gao et al., 2022; Ruan et al., 2022). Despite the widespread use of chemotherapy, its effectiveness is frequently compromised by the unique physiological constraints of hypoxia, often leading to a trade-off between severe side effects and inadequate therapeutic response (Chen et al., 2025; Liu et al., 2025; van den Boogaard et al., 2022). Therefore, exploring effective approaches to alleviate tumor hypoxia is imperative for advancing HCC management.

Natural products have emerged as a reservoir of potent anticancer agents due to their diverse biological activities (Huang et al., 2025; Ranamalla et al., 2025; Veerabomma et al., 2025). Shikonin (SK), a prominent naphthoquinone derived from *Lithospermum erythrorhizon*, exhibits robust antitumor efficacy. SK targets pyruvate kinase M2 (PKM2), a key enzyme regulating glycolytic metabolism (Wang et al., 2025b). Also, SK exerts cytotoxicity through oxygen-coupled redox cycling, utilizing oxygen to generate superoxide anions (Zhang et al., 2022; Yang et al., 2014). Glycolysis provides adenosine triphosphate (ATP) and metabolic intermediates that fuel tumor growth (Yang et al., 2022; Zhang et al., 2023; Zhou et al., 2022). SK disrupts glycolytic output by inhibiting PKM2, thereby depleting ATP and essential biosynthetic precursors required for rapid tumor proliferation (Jingtai et al., 2023; Park et al., 2022). However, the therapeutic efficacy of SK is often compromised by hypoxia. Specifically, hypoxia triggers the upregulation of glycolytic enzymes and survival signaling pathways, which can counteract the inhibitory effect of SK on PKM2. The catalase (CAT), an endogenous enzyme, can decompose hydrogen peroxide (H_2_O_2_) into water and oxygen (O_2_) to alleviate hypoxia (Li et al., 2024a; Wan et al., 2022; Yang et al., 2023). The CAT-catalyzed in situ oxygen generation effectively alleviates tumor hypoxic microenvironment and thereby enhances the sensitivity of tumor cells to the chemotherapeutic agent (Liu et al., 2024; Yang et al., 2025). Also, the CAT-catalyzed oxygen generation disrupt the robust glycolytic dependency of hypoxic tumors, leading to a more profound inhibition of tumor progression. The poor aqueous solubility of SK and the intrinsic instability of CAT necessitate advanced delivery systems to co-deliver and protect these agents while improving tumor accumulation and therapeutic performance.

The rapid development of nanotechnology has paved the way for efficient drug delivery systems to improve the efficacy and safety of cancer therapy (Chiang et al., 2024; Huynh et al., 2025a; Huynh et al., 2025b; Lien et al., 2026; Masoumi Shahrbabak et al., 2025). Nanomaterial-based drug delivery systems can effectively circumvent the limitations of free drugs by improving their solubility and enabling stimuli-responsive release, thereby maximizing tumor accumulation through the enhanced permeability and retention (EPR) effect (Hu et al., 2023). Among various nanocarriers, polydopamine (PDA) has emerged as a versatile candidate due to its exceptional biocompatibility, robust near-infrared (NIR) photothermal conversion, and abundant functional groups for drug loading and surface tailoring (Li et al., 2025; Tang et al., 2024; Wei et al., 2024). Beyond its role as a stable nanoplatform, PDA-mediated photothermal therapy (PTT) offers precise spatial control and often acts synergistically with chemotherapy to overcome drug resistance (Lu et al., 2021; Ma et al., 2022; Xu et al., 2024). Furthermore, a series of glycoconjugated nanoparticles has been previously established to target hepatocytes via asialoglycoprotein receptor (ASGPR) by our group (Hu et al., 2022; Wang et al., 2025a; Xu et al., 2025). These targeted platforms have demonstrated superior HCC affinity and potent anti-tumor activity in vitro and in vivo.

**Scheme 1:**
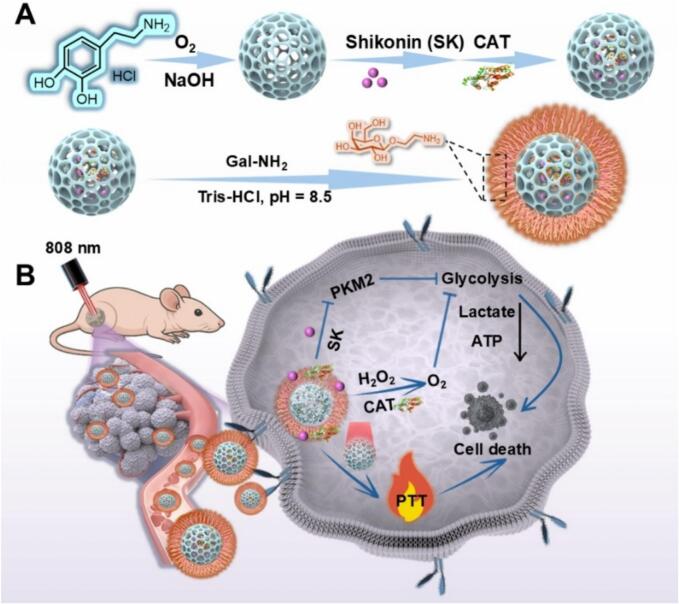
(A) Schematic illustration of the preparation of SC@PDA-Gal. (B) Schematic illustration of chemo-photothermal therapy mediated by SC@PDA-Gal.

In this study, a multifunctional polydopamine-based nanomedicine (SC@PDA-Gal) was developed for synergistic HCC therapy (Scheme 1). This nanomedicine achieved enhanced efficacy by co-delivering SK and CAT, which simultaneously inhibited glycolysis and alleviated hypoxia, thereby sensitizing tumor cells to metabolic exhaustion. These agents were efficiently loaded onto PDA nanocarriers, while PDA provided efficient photothermal conversion under NIR irradiation to further enhance therapy. To ensure precision, surface functionalization with Galactose (Gal) facilitated ASGPR-mediated targeting, maximizing tumor accumulation to minimize toxicity. Overall, this integrated nanomedicine offers a potent and bio-safe approach for oxygen-augmented chemo-photothermal therapy, providing a promising direction for HCC management.

## 2 Materials and methods

### 2.1 Materials

Shikonin (SK) was purchased from Daosifu (Nanjing, China). catalase (CAT) was purchased from Yuanye (Shanghai, China). Rhodamine B (RhB) was purchased from J&K (Shanghai, China). ATP and lactate Content Assay Kit were purchased from Beyotime (Shanghai, China).

### 2.2 Preparation of PDA-based nanocomposites

PDA was prepared according to a previous report (Du et al., 2021). Briefly, SK (10 mg) was dissolved in methanol (2 mL), and PDA (5 mg) was dispersed in deionized water (2 mL). The two solutions were mixed and stirred in the dark for 12 h. The product was collected by washing and lyophilization to obtain SK@PDA. SK@PDA and CAT were mixed in PBS (pH 7.0) at a mass ratio of 1:2 (*w*/w) and stirred in the dark for 12 h, followed by washing and lyophilization to yield SC@PDA. SC@PDA and amine-modified Gal were reacted in Tris buffer (10 mM, pH 8.5) at a mass ratio of 1:2 (w/w) for 12 h in the dark, then washed and lyophilized to afford SC@PDA-Gal.

### 2.3 In vitro photothermal effect and evaluation of enzymatic activity

To evaluate the photothermal performance, SC@PDA-Gal aqueous dispersions at different concentrations (0, 100, 200, and 500 μg mL^−1^) and PDA solution (100 μg mL^−1^) were placed in centrifuge tubes and irradiated with an 808 nm NIR laser at 1.5 W cm^−2^ for 300 s. The temperature was recorded in real time. For the power-dependent study, SC@PDA-Gal dispersion (100 μg mL^−1^) was irradiated under different power densities (0, 1.0, 1.5, and 2.0 W cm^−2^) for 5 min. Temperature changes during the above processes were real-time monitored and recorded using a digital thermometer.

To assess the activity of CAT in the particles, H_2_O_2_ was first added into water to prepare an H_2_O_2_ solution. Subsequently, SC@PDA-Gal composites were added to this H_2_O_2_ solution, and the generation of bubbles in the solution was observed.

### 2.4 In vitro therapeutic evaluation

Live/dead staining was performed using a Calcein-AM/PI kit to evaluate the therapeutic effect of SC@PDA-Gal. SMMC-7721 cells were seeded in 48-well plate at a density of 2 × 10^4^ cells per dish and incubated for 24 h. The medium was then replaced with 200 μL of fresh medium containing SC@PDA-Gal at different concentrations, or SC@PDA-Gal loaded with SK (1 μg mL^−1^), and the cells were incubated for another 24 h. Subsequently, cells in the designated groups were irradiated with an 808 nm laser (1.5 W cm^−2^, 5 min). Free SK (1 μg mL^−1^) was used as a control. After treatment, the cells were stained with Calcein-AM/PI working solution for 15 min and immediately imaged.

### 2.5 Biodistribution of SC@PDA-Gal

All animal procedures were approved by the Experimental Animal Ethics Committee of Jiangnan University (JN. No20241230b0720630; Wuxi, Jiangsu, China) and were performed in accordance with relevant guidelines and regulations for the care and use of laboratory animals.

SC@PDA-Gal and SC@PDA were fluorescently labeled with rhodamine B (RhB). Female BALB/c nude mice (five weeks old, 18–20 g) were used to establish a subcutaneous tumor model. Briefly, C5WN1 cells (2 × 10^6^ cells mL^−1^, 100 μL) were subcutaneously injected into the right flank (above the right hind limb). Tumor size was measured every other day, and tumor volume was calculated as: V = 0.5 × L × W^2^, where L and W represent the tumor length and width, respectively.

When the tumor volume reached ∼100 mm^3^, mice were intravenously injected with SC@PDA-Gal-RhB and SC@PDA-RhB. In vivo fluorescence imaging was performed using an IVIS imaging system (IVIS Lumina LT Series III, PerkinElmer, USA) at predetermined time points. After imaging, mice were euthanized, and major organs (heart, liver, spleen, lung, and kidney) as well as tumor tissues were harvested for ex vivo fluorescence analysis.

### 2.6 In vivo antitumor efficiency

C5WN1 tumor-bearing BALB/c nude mice were established as described above. When the tumor volume reached approximately 50 mm^3^, the mice were used for in vivo antitumor therapy. The tumor-bearing mice were randomly assigned to eight groups (*n* = 5 per group) and intravenously injected (tail vein) with different formulations: PBS, PDA, PDA + NIR, SK, SK@PDA, SC@PDA, SC@PDA-Gal, and SC@PDA-Gal + NIR. For the groups receiving laser irradiation, the tumor region was exposed to an 808 nm NIR laser (1.5 W cm^−2^) for 5 min after each injection. Treatments were administered once every other day for a total of four injections. Tumor size and body weight were measured and recorded every other day using digital calipers and an electronic balance, respectively. On day 14 after the first treatment, mice were euthanized, and tumors were excised, photographed, and collected. Major organs (heart, liver, spleen, lung, and kidney) and tumor tissues were harvested for histological analysis and subjected to hematoxylin and eosin (H&E) staining.

### 2.7 Statistical analysis

The experiments were repeated at least three times. The statistical significance of data was determined by one-way analysis of variance (ANOVA) with GraphPad Prism 10.0. Results were shown as mean ± standard deviation (mean ± SD) (*n* ≥ 3).

## 3 Results and discussions

### 3.1 Fabrication and characterization of SC@PDA-Gal

**Fig. 1:**
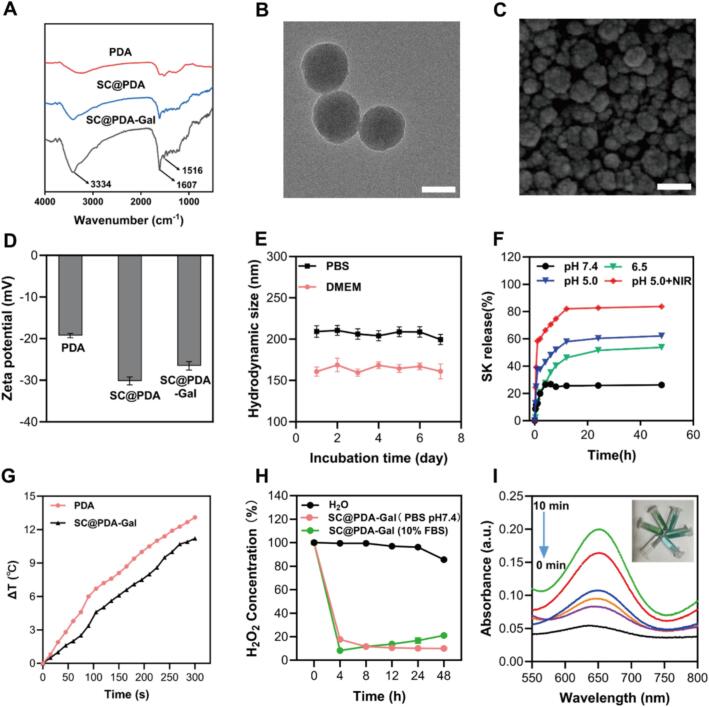
Characterizations of SC@PDA-Gal. (A) FT-IR spectra of PDA, SC@PDA-Gal and SC@PDA-Gal. (B) TEM image of SC@PDA-Gal. Scale bar: 200 nm. (C) SEM image of SC@PDA-Gal. Scale bar: 200 nm. (D) Zeta potential of indicated NPs. Mean ± SD (*n* = 3). (E) Long-term hydrodynamic size stability of SC@PDA-Gal. Mean ± SD (n = 3). (F) Release of SK from SC@PDA-Gal under different conditions. Mean ± SD (n = 3). (G) Temperature variation curves of the aqueous dispersions of PDA and SC@PDA-Gal exposed to the 808 nm laser (1.5 W/cm^2^, 5 min). (H) Decomposition of H_2_O_2_ (1.0 mM) with and without SC@PDA-Gal (preincubation in PBS and DMEM with 10% FBS). (I) Changes in absorbance spectra of TMB mixed with SC@PDA-Gal incubated into the 1.0 mM H_2_O_2_ solution for different time.

PDA were synthesized following a reported protocol (Du et al., 2021). SK, a natural naphthoquinone compound with known anticancer activity, was loaded onto PDA via π–π stacking (Lv et al., 2022). CAT was immobilized on the surface owing to the adhesive property of PDA, yielding SC@PDA. An ASGPR-targeting galactose molecule (amine-modified galactose, Gal) was synthesized using an established procedure38 and covalently conjugated with SC@PDA to generate SC@PDA-Gal, which exhibited improved stability and hepatocyte-targeting capability. Fourier transform infrared (FTIR) spectroscopy revealed the SC@PDA-Gal structure (Fig. 1A). The absorption band at 1607 cm^−1^ was attributed to the C=O stretching vibration of SK (Ma et al., 2022), while the band at 1516 cm^−1^ corresponded to the C=C stretching vibration of CAT (Xu et al., 2024). And the efficient grafting of Gal onto SC@PDA was validated by the appearance of an enhanced band at 3334 cm^−1^ assigned to O—H stretching vibration (Wang et al., 2025a). The loading content of Gal in SC@PDA-Gal was determined to be 1.16 ± 0.01% by High-Performance Liquid Chromatography (HPLC) analysis. The UV–vis absorption spectroscopy revealed the successful loading of SK into SC@PDA-Gal. The characteristic peak at 517 nm of SK was detected for SC@PDA-Gal compared with PDA (Fig. S1).

Transmission and scanning electron microscopy (TEM and SEM) analyses showed that SC@PDA-Gal possessed a well-defined spherical morphology (Fig. 1B and C). The zeta potential was measured by dynamic light scattering (DLS). PDA showed a zeta potential of −19.26 ± 0.50 mV (Fig. 1D), and the loading of SK and CAT significantly decreased the zeta potential of SC@PDA to −30.17 ± 0.96 mV. The zeta potential of SC@PDA-Gal increased to −26.53 ± 1.00 mV after Gal coating, owing to the presence of positively charged amino termini. The hydrodynamic diameter and size distribution were also measured. PDA and SC@PDA-Gal showed average diameters of 184.4 ± 1.15 nm (PDI = 0.069) and 211.1 ± 0.12 nm (PDI = 0.026), respectively (Fig. S2 and S3). The stability of SC@PDA-Gal was evaluated in PBS (pH 7.4) and DMEM containing 10% FBS for 7 days (Fig. 1E). The particle showed negligible size variation, indicating excellent stability under physiological conditions. Optimized DLC and DEE were achieved when PDA, SK, and CAT were mixed in a (1:2):2 (*w*/w/w) ratio (Table S1). The drug loading capacity (DLE) and drug encapsulation efficiency (DLE) of SK were determined to be 26.02 ± 0.12% and 51.89 ± 0.09%, respectively. And the DLE and DEE of CAT were measured as 4.50 ± 0.12% and 18.00 ± 0.48%, respectively. The in vitro SK release from SK@PDA-Gal was assessed at pH 7.4, 6.5 and 5.0, and the effect of NIR irradiation was examined at pH 5.0. The cumulative release at pH 5.0 reached 83.70 ± 0.13% at 48 h under NIR irradiation (Fig. 1F), whereas it was 62.15 ± 0.10% without NIR. At pH 6.5, a tumor-relevant mildly acidic condition, the cumulative release reached 54.2 ± 0.18% at 48 h. By contrast, only 26.15 ± 0.03% of SK was released at pH 7.4 over 48 h. These findings indicate a pH-responsive and NIR-enhanced release profile, supporting more efficient drug release under acidic conditions.

### 3.2 Photothermal effect and enzyme activity of SC@PDA-Gal

The photothermal effects of SC@PDA-Gal was evaluated by monitoring temperature changes under 808 nm laser irradiation. After 5 min of irradiation at 1.5 W cm^−2^, the temperature of 100 μg mL^−1^ PDA and SC@PDA-Gal solution both increased by more than 9 °C (Fig. 1G). Moreover, the temperature rise of SC@PDA-Gal was positively correlated with both its concentration and the power density of the NIR laser (Fig. S4). These results demonstrated that SC@PDA-Gal retained the intrinsic photothermal properties of PDA and the incorporation of SK, CAT, and galactose did not compromise the photothermal conversion efficiency of PDA. To investigate the enzyme activity of SC@PDA-Gal, H_2_O_2_ was added into the composites suspension and reacted for different time. Upon adding SC@PDA-Gal to the H_2_O_2_ solution, noticeable bubbling occurred, indicating O_2_ generation catalyzed by CAT in SC@PDA-Gal (Fig. S5). Only 15% of H_2_O_2_ remained after incubation with SC@PDA-Gal for 48 h according to the results of titanium sulfate colorimetric assay (Fig. 1H), verifying the decomposition of H_2_O_2_ by CAT in SC@PDA-Gal. The catalytic activity was evaluated in DMEM containing 10% FBS. SC@PDA-Gal retained robust H_2_O_2_ decomposition capability in the serum-containing environment, indicating that the nanoplatform effectively maintains CAT activity under physiologically relevant conditions. 3,3′,5,5′-tetramethylbenzidine (TMB) reacts with H_2_O_2_ to produce a blue-colored product in the presence of horseradish peroxidase (HRP). After adding H_2_O_2_ to SC@PDA-Gal, the absorbance of TMB at 652 nm gradually decreased over time, accompanied by a visible fading of the blue color (Fig. 1I). These results collectively demonstrated that the SC@PDA-Gal retain the catalytic activity of CAT and catalyze the decomposition of H_2_O_2_ into O_2_.

### 3.3 In vitro targeting ability of SC@PDA-Gal

**Fig. 2:**
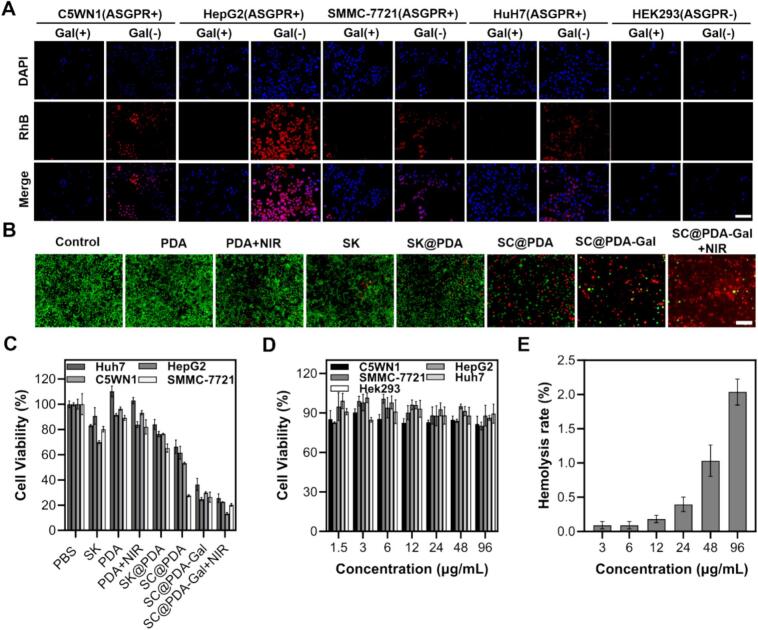
(A) Fluorescence microscopic images of C5WN1, HepG2, SMMC-7721, Huh7 and HEK293 cells incubated with SC@PDA-Gal with or without free galactose (1 mmol/L) competition. Scale bar: 25 μm. (B) Calcein-AM/propidium iodide double staining of SMMC-7721 cells after treatment with different agents. Scale bar: 200 μm. (C) In vitro growth inhibition of Huh7, HepG2, C5WN1, and SMMC-7721 cells treated with various agents for 48 h. Mean ± SD (*n* = 3). (D) Viability of C5WN1, HepG2, SMMC-7721, Huh7 and HEK293 cells treated with PDA at various concentrations for 48 h. Mean ± SD (n = 3). (E) Hemolysis test results of SC@PDA-Gal-RhB at different concentrations.

RhB-labeled SC@PDA-Gal was used for targeted tracing and denoted as SC@PDA-Gal-RhB. The targeting specificity of SC@PDA-Gal in vitro was evaluated by fluorescence microscopy and flow cytometry analysis. ASGPR-high-expressing cells (C5WN1, HepG2, Huh7, and SMMC-7721, designated as ASGPR^+^ cells) were incubated with SC@PDA-Gal-RhB for 24 h. ASGPR-low-expressing HEK293 cells were served as negative controls (ASGPR^-^). Strong RhB fluorescence was observed in ASGPR^+^ cells. In contrast, almost no fluorescence was detected in ASGPR- cells or in ASGPR^+^ cells pretreated with free galactose as a competitive inhibitor (Fig. 2A). These results indicated that the galactose-modified nanocomposites can specifically recognize ASGPR+ cells and promote cellular internalization. Flow cytometry was further conducted to verify the targeting effect. A marked decrease in fluorescence intensity was observed in ASGPR^+^ cells at galactose-competition group, whereas no significant change occurred in ASGPR^-^ cells (Fig. S6). These results demonstrated the specific binding and mediated internalization of SC@PDA-Gal in ASGPR^+^ cells.

### 3.4 In vitro cytotoxicity and biosafety of SC@PDA-Gal

Live/dead staining was performed to visualize the treatment-induced cytotoxicity. All groups included: PBS (vehicle control), PDA (blank carrier), PDA + NIR (PDA with 808 nm irradiation), SK (free SK), SK@PDA (SK-loaded PDA), SC@PDA (SK/CAT-loaded PDA), SC@PDA-Gal (galactose-modified SC@PDA), and SC@PDA-Gal + NIR (SC@PDA-Gal with 808 nm irradiation). Minimal PI-positive signals in the PBS and PDA groups (Fig. 2B), and PDA + NIR induced moderate cytotoxicity attributable to the photothermal effect. Notably, SC@PDA-Gal + NIR induced extensive cell death, evidenced by a dominant red signal with minimal green fluorescence.

The in vitro antitumor efficacy of SC@PDA-Gal was then quantified by 3-(4,5-dimethylthiazol-2-yl)-2,5-diphenyltetrazolium bromide (MTT) assay in four HCC cell lines (C5WN1, Huh7, HepG2, and SMMC-7721). Free SK showed moderate cytotoxicity, with cell viabilities of 69.98 ± 1.24% to 90.75 ± 6.59% (Fig. 2C), and a comparable effect was observed for SK@PDA (65.25 ± 3.26% to 84.00 ± 3.88%), indicating limited therapeutic benefit from non-targeted loading. Co-loading SK and CAT in SC@PDA significantly enhanced cytotoxicity (27.57 ± 0.86% to 63.30 ± 5.28%). This inhibitory effect was further amplified by SC@PDA-Gal (24.78 ± 1.29% to 36.46 ± 4.78%), underscoring the advantages of Gal-mediated ASGPR recognition and receptor-mediated endocytosis. Notably, SC@PDA-Gal + NIR produced the strongest effect, decreasing the viability of all four cell lines to below 30%, highlighting the superior efficacy of the targeted chemo-photothermal strategy. Moreover, SC@PDA-Gal reduced cell viability in a concentration-dependent manner (Fig. S7). To quantitatively evaluate the interaction between chemotherapy and phototherapy, the Bliss independence analysis was employed (Meyer et al., 2020). The combination treatment (SC@PDA-Gal + NIR) exhibited enhanced anti-tumor activity compared to monotherapies (Fig. 2C). Specifically, in C5WN1 cells, SC@PDA-Gal + NIR yielded a positive synergy scores (ΔI) of 11.3%, indicating a synergistic chemo-photothermal interaction. Collectively, these data confirmed the potent synergistic antitumor activity of SC@PDA-Gal in vitro.

The safety profile of blank nanomaterials is a key criterion for subsequent biological applications. The viabilities of C5WN1, HepG2, Huh7, SMMC-7721, and HEK293 cells remained above 80% after incubation with PDA at 96 μg·mL^−1^ (Fig. 2D), indicating minimal intrinsic cytotoxicity. In addition, the hemocompatibility of SC@PDA-Gal was evaluated using a red blood cell hemolysis assay. The hemolysis rate remained below 2% even at concentrations up to 96 μg·mL^−1^ (Fig. 2E), suggesting a low risk of erythrocyte membrane damage. Collectively, these results demonstrated that SC@PDA-Gal exhibit good biocompatibility and is suitable for further in vivo studies.

### 3.5 In vitro mechanism for inhabiting tumor glycolysis of SC@PDA-Gal

**Fig. 3:**
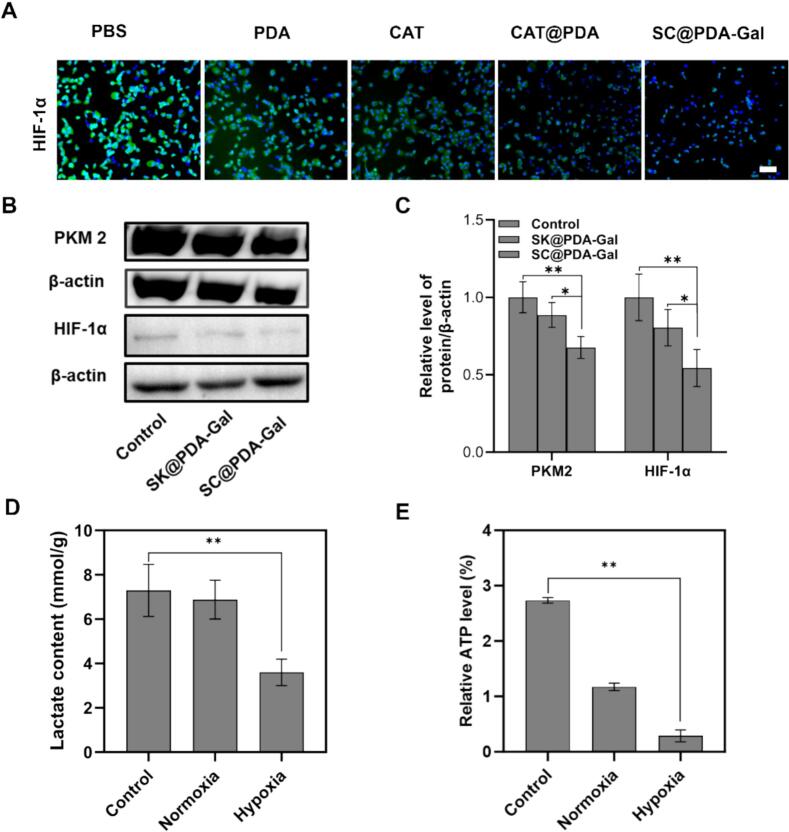
(A) Immunofluorescent staining of HIF-1α in C5WN1 cells after various treatments. Blue fluorescence is DAPI from nuclei, green comes from HIF-1α. Scale bar: 200 μm. (B) Western blotting detection of PKM2 and HIF-1α in C5WN1 cells following treatment with Control, SK@PDA-Gal, and SC@PDA-Gal. (C) Semiquantitative analysis of (B) assay. *p < 0.05. Mean ± SD (*n* = 3). (D) Detection of cellular lactate and (E) ATP levels treated with SC@PDA-Gal under normoxic and hypoxic conditions. **p < 0.01. Mean ± SD (n = 3). (For interpretation of the references to color in this figure legend, the reader is referred to the web version of this article.)

To elucidate whether SC@PDA-Gal modulates hypoxia and glycolysis in tumor cells, HIF-1α and key glycolytic markers were examined. Immunofluorescence staining showed that HIF-1α fluorescence was weakest in the SC@PDA-Gal group among all treatments (Fig. 3A), indicating a pronounced alleviation of cellular hypoxia. Notably, SC@PDA-Gal induced a more evident reduction in HIF-1α than CAT alone, suggesting that the constructed nanodrug enhances the hypoxia-relieving effect beyond single-enzyme treatment. Consistently, it was further confirmed that SC@PDA-Gal downregulated HIF-1α protein expression by western blot analysis (Fig. 3B and C). Given that catalase (CAT) can decompose excess H_2_O_2_ to generate O_2_, the decreased HIF-1α level is in line with improved oxygenation and weakened hypoxia fluorescence signaling.

The potential of SC@PDA-Gal to suppress tumor glycolysis was subsequently investigated. It showed a marked reduction in the expression of PKM2 in western blot analysis, a rate-limiting enzyme in tumor glycolysis, following SC@PDA-Gal treatment (Fig. 3B and C). Given that SK is a known inhibitor of PKM2 (Rihan and Sharma, 2024), this downregulation is consistent with SK-mediated disruption of glycolytic activity (Xiang et al., 2025). The intracellular lactate and ATP levels were further quantified. Under hypoxic conditions, SC@PDA-Gal significantly attenuated lactate production (3.60 ± 0.60 mmol/g, 51% decrease vs. control) and decreased ATP content (0.29 ± 0.11%, 89% decrease vs. control) (Fig. 3D and E), indicating a compromised glycolytic output. Collectively, these results suggest that SC@PDA-Gal exerts a dual metabolic intervention: it simultaneously alleviates hypoxia (via CAT-mediated O_2_ generation and subsequent HIF-1α suppression) and restrains glycolysis (via PKM2 downregulation), thereby weakening the metabolic support for tumor cell proliferation.

### 3.6 In vivo biodistribution of SC@PDA-Gal

**Fig. 4:**
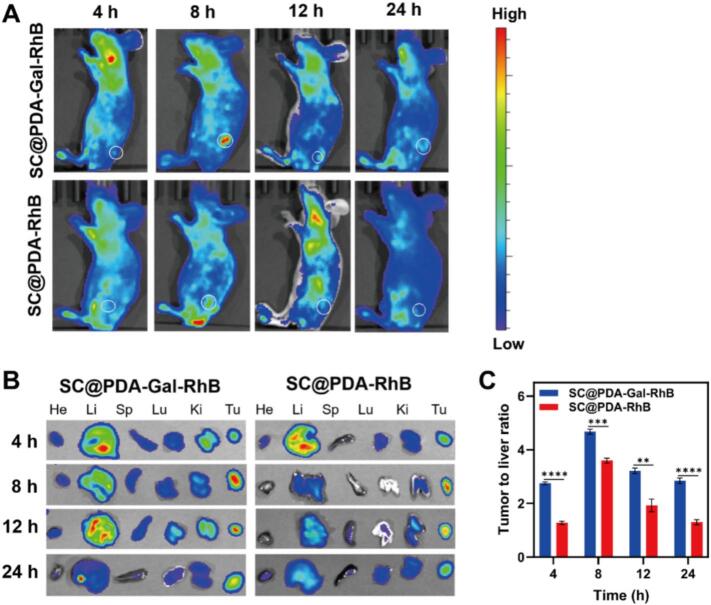
(A) In vivo fluorescence imaging results of rhodamine B (RhB) at different time points after intravenous injection of SC@PDA-Gal-RhB and SC@PDA-RhB formulations via the tail vein. (B) Representative in vivo fluorescence images of RhB in harvested organs (He: Heart; Li: Liver; Sp: Spleen; Lu: Lung; Ki: Kidney; Tu: Tumor) after different injection times. (C) Semi-quantitative analysis of the tumor-to-liver fluorescence intensity ratios (T-L ratios) calculated from the ex vivo images in (B). **p < 0.01, ***p < 0.001, ****p < 0.0001. Mean ± SD (*n* = 3).

In vivo biodistribution was assessed in subcutaneous C5WN1 tumor-bearing mice using fluorescence imaging to validate the targeting capability of SC@PDA-Gal (Fig. 4A). RhB labeling was used to confer fluorescence tracing capability for both the targeted and non-targeted formulations, yielding SC@PDA-Gal-RhB (targeted; RhB labeled SC@PDA-Gal) and SC@PDA-RhB (non-targeted; RhB labeled SC@PDA). S SC@PDA-Gal-RhB exhibited a clearly enhanced tumor-associated fluorescence signal starting at 8 h post-injection and reaching a maximum at 12 h, indicating efficient tumor accumulation mediated by Gal modification. Notably, the tumor fluorescence remained prominent at 18 h and gradually declined by 24 h, suggesting prolonged tumor retention. In contrast, SC@PDA-RhB distributed broadly throughout the body after intravenous injection, with no obvious enrichment at the tumor site.

Ex vivo imaging of excised tumors and major organs at the indicated time points further supported the in vivo observations (Fig. 4B). In the SC@PDA-Gal-RhB group, strong fluorescence was mainly detected in the tumor, with additional signals in the liver and kidneys, which may be attributed to reticuloendothelial system (RES) uptake in the liver and subsequent hepatic and/or renal clearance. To semi-quantitatively evaluate the targeting specificity, the Tumor-to-Liver (T/L) ratios were calculated based on the fluorescence intensity (Fig. 4C). The T/L ratio of the SC@PDA-Gal-RhB remained consistently above 2.5 throughout the 24 h period, reaching a peak of approximately 4.6 at 8 h. This was significantly higher than that of the SC@PDA-RhB at all time points (*p* < 0.05). These high T/L ratios (> 1) provided evidence of tumor-preferential accumulation relative to hepatic accumulation at the organ level. This tumor-preferential distribution is consistent with the combined contributions of ASGPR-mediated active uptake and passive tumor retention (EPR effect). (Wang et al., 2023; Du et al., 2021). Collectively, these results demonstrated that SC@PDA-Gal achieved enhanced tumor-targeted delivery and prolonged retention in vivo, providing a favorable basis for subsequent antitumor evaluation.

### 3.7 In vivo anti-tumor efficacy of SC@PDA-Gal

The in vivo therapeutic efficacy of SC@PDA-Gal was further evaluated in subcutaneous C5WN1 tumor-bearing mice over a two-week treatment period. Mice were randomly assigned to eight groups receiving: saline, PDA (blank carrier), PDA + NIR (PDA with NIR), SK (free SK), SK@PDA (SK-loaded PDA), SC@PDA (SK/CAT co-loaded PDA), SC@PDA-Gal (galactose-modified SC@PDA), and SC@PDA-Gal + NIR (SC@PDA-Gal with NIR). Mice were intravenously injected every 2 days, followed by 808 nm irradiation for 5 min at 12 h post-injection in the designated NIR groups. Tumor volumes were recorded at predetermined time points throughout the treatment.

**Fig. 5:**
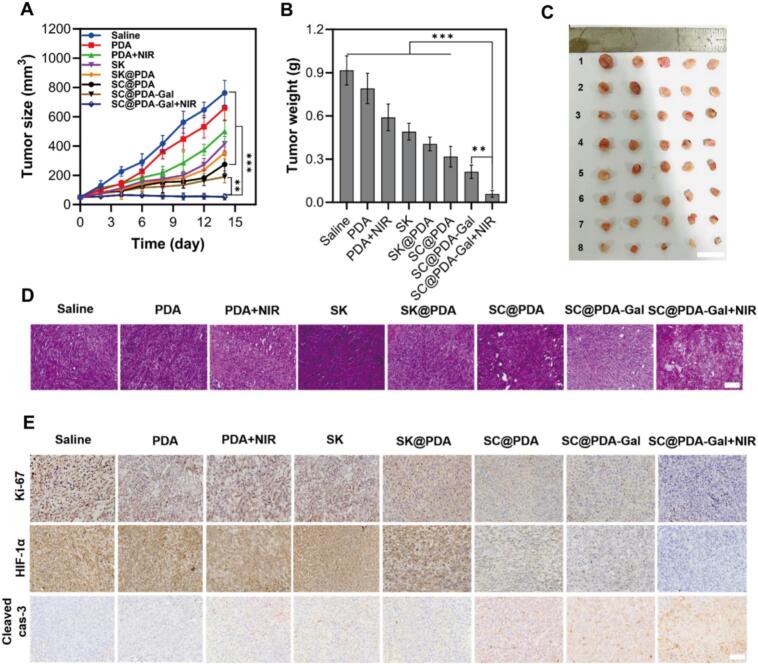
(A) Growth curves of tumors in the mice treated with different groups. Mean ± SD, *n* = 5. *p < 0.05, **p < 0.01, ***p < 0.001. (B) Tumor weights collected on day 14. Mean ± SD, n = 5. *p < 0.05, **p < 0.01, ***p < 0.001. (C) Photographs of dissected tumors in each group. Scale bar: 1 cm. (D) Hematoxylin and eosin (H&E) staining results of tumor tissues after different treatments. Scale bar: 50 μm. (E) Immunohistochemical (IHC) staining images of Ki67, HIF-1α and Cleaved caspase 3 in tumor tissues from each group. Scale bar: 50 μm.

Tumors in the SC@PDA-Gal + NIR group showed minimal growth and remained at 915.90 ± 101.39 mm^3^ by the end of the study, whereas the saline control reached 58.79 ± 2.35 mm^3^ (Fig. 5A). PDA alone showed no obvious therapeutic effect and exhibited a tumor growth profile comparable to the saline group (Fig. 5B). Free SK and PDA + NIR produced moderate tumor suppression, with tumor inhibition rates (TIRs) of 45.75 ± 9.97% and 34.90 ± 11.84%, respectively, indicating contributions from SK chemotherapy and photothermal treatment. The SC@PDA group exhibited a higher TIR (64.41 ± 12.32%), suggesting that co-loading SK and CAT enhanced antitumor efficacy. Further improvement was achieved with SC@PDA-Gal (TIR of 76.57 ± 5.43%), likely due to Gal-mediated targeting effect. Notably, SC@PDA-Gal + NIR yielded the strongest tumor suppression and the highest TIR (93.44 ± 2.86%, *P* < 0.001) among all groups (Fig. 5C). SC@PDA-Gal + NIR produced a higher TIR than free sorafenib (clinically approved first-line drugs) reported previously in the same C5WN1 model (TIR of 39%, Hu et al., 2022), highlighting the translational potential of nanomedicine-enabled delivery strategies for HCC.

The hematoxylin and eosin (H&E) staining of tumor sections revealed the most extensive tumor necrosis in the SC@PDA-Gal + NIR group (Fig. 5D), which is consistent with the tumor inhibition results. Immunohistochemical analysis (Fig. 5E) and corresponding quantitative evaluation (Fig. S8) further showed that SC@PDA-Gal-based treatments markedly reduced the expression of Ki-67, suggesting suppressed tumor proliferation. The HIF-1α positive rate significantly decreased from 88.1 ± 3.2% in the saline group to 17.8 ± 2.8% in the SC@PDA-Gal + NIR group. This approximately 70% reduction in the hypoxia marker provides evidence consistent with effective intratumoral hypoxia relief. And the SC@PDA-Gal + NIR group exhibits markedly increased cleaved caspase-3 positivity, indicating pronounced tumor cell apoptosis after treatment. Overall, these findings supported a multi-modal synergistic effect of the integrated SK/CAT/photothermal strategy.

Throughout the treatment, mouse body weights remained stable across all groups (Fig. S9), indicating no apparent systemic toxicity. H&E staining of major organs (heart, liver, spleen, lung, and kidney) showed no evident pathological abnormalities (Fig. S10). In addition, serum biochemistry markers (ALT, AST, ALP, CREA, and BUN) remained within normal ranges, suggesting preserved hepatic and renal function (Fig. S11). Routine blood indices (WBC, RBC, HGB, and PLT) were also within normal limits, indicating no obvious hematological toxicity. Collectively, these results support the favorable in vivo biosafety and biocompatibility of SC@PDA-Gal.

## 4 Conclusion

In conclusion, this study successfully developed SC@PDA-Gal for the chemo-photothermal therapy of HCC. Both in vitro and in vivo studies demonstrated that SC@PDA-Gal effectively suppressed tumor glycolysis via PKM2 inhibition, significantly reducing glycolytic output. Concurrently, CAT-mediated in situ oxygen generation alleviated hypoxia and downregulated HIF-1α, thereby sensitizing hypoxic tumor cells to SK-mediated cytotoxicity. Combined with PDA-mediated hyperthermia, this system fostered a robust chemo-photothermal synergy that markedly amplified overall efficacy. In addition, SC@PDA-Gal facilitated efficient tumor accumulation and achieved enhanced therapeutic outcomes. Future work will further evaluate targeting performance and therapeutic efficacy in orthotopic HCC models to better recapitulate the intrahepatic microenvironment and facilitate potential clinical translation. Thus, SC@PDA-Gal represents a promising hypoxia-adaptive nanomedicine for advanced HCC therapy.

## CRediT authorship contribution statement

**Xiang Wang:** Writing – original draft, Visualization, Investigation. **Yihan Ma:** Writing – original draft, Validation, Investigation. **Le Wang:** Investigation. **Hengrui Li:** Data curation. **Miao Qin:** Investigation. **Ruonan Sun:** Validation. **Jing Hu:** Writing – review & editing, Supervision, Methodology, Funding acquisition, Conceptualization.

## Declaration of competing interest

The authors declare that they have no known competing financial interests or personal relationships that could have appeared to influence the work reported in this paper.

## Data Availability

Data will be made available on request.

## Appendix A Supplementary data

Supplementary material Image 1
