# Supplementary material for: Hypoxia-relieving and glycolysis-disrupting polydopamine nanomedicine for synergistic chemo-photothermal therapy of hepatocellular carcinoma
Source: Int J Pharm X. 2026 Feb 18;11:100506. doi: 10.1016/j.ijpx.2026.100506 (PMC12962110; doi:10.1016/j.ijpx.2026.100506)
Supplement: Supplementary file 1 — Supplementary material [file mmc1.docx]

**Supplementary information**

**Hypoxia-Relieving and Glycolysis-Disrupting Polydopamine Nanomedicine for Synergistic Chemo-Photothermal Therapy of Hepatocellular Carcinoma**

**Xiang Wang^a,c,#^, Yihan Ma^a,#^, Le Wang^b^, Hengrui Li^a^, Miao Qin^a^, Ruonan Sun^a^, Jing Hu^a,d*^**

^a^Wuxi School of Medicine, Jiangnan University, Wuxi, P.R. China.

^b^School of Biotechnology and Key Laboratory of Carbohydrate Chemistry and Biotechnology of Ministry of Education, Jiangnan University, Wuxi, 214122, China.

^c^School of Food Science and Technology, Jiangnan University, Wuxi 214122, China.

^d^Institute of Future Food Technology, JITRI, No.19 Wenzhuang Road, Yixing 214200, China.

*Corresponding authors: Jing Hu, Ph.D., Professor, Wuxi School of Medicine, Jiangnan University, Wuxi 214122, China; Institute of Future Food Technology, JITRI, Yixing 214200, China; orcid.org/0000-0003-3288-7067; Email: hujing@jiangnan.edu.cn.

^#^These authors contributed equally to this work.

**Materials and Methods**

Dopamine hydrochloride, and Tris were purchased from J&K (Shanghai, China). Calcein-AM/PI Cell Viability/Cytotoxicity Assay Kit, Dulbecco’s Modified Eagle Medium (DMEM, Gibco), basicRPMI1640 medium, fetal bovine serum (FBS, Gibco) and 0.25 % tryp­sin–EDTA were purchased from Thermo Fisher Scientific (Shanghai,China). HIF-1α Antibody, PKM2 Antibody, Ki-67Antibody, and Beta Actin Antibody were purchased from Med Chem Express (Shanghai, China).

**Characterizations of** **PDA-based nanocomposites**

The hydrodynamic size and zeta potential of PDA and SC@PDA-Gal were were measured by Nano ZS ZEN360. The morphology of NPs was analyzed by transmission electron microscopy (TEM) and scanning electron microscopy (SEM). The chemical characteristic peaks of each component were analyzed by Fourier transform infrared (FT-IR) spectrometer and ultraviolet-visible spectrophotometer (UV) to characterize the synthesis of SC@PDA-Gal successfully.

**Quantification of Galactose Modification**

The galactose (Gal) loading content was determined by High-Performance Liquid Chromatography (HPLC). Briefly, the supernatant containing unreacted Gal was collected after centrifugation. The concentration of free Gal was analyzed using an HPLC system. Based on the standard curve (Y = 9162X + 1148, R^2^ > 0.99, Y represents the peak area and X represents the concentration of galactose), the mass of conjugated Gal was calculated by subtracting the unreacted Gal from the total input. The loading content was calculated as follows: Loading content (%) = (Mass of conjugated Gal/ Total mass of nanoparticles) × 100%.

**Stability of SC@PDA-Gal**

SC@PDA-Gal was dissolved in PBS buffer (pH 7.4) and medium (DMEM containing 10% fetal bovine serum) respectively. The size changes of the nanocomposite in the two solutions within 7 days were measured using a Nano ZS ZEN360 instrument, so as to evaluate the stability of the nanocomposite in different solution environments.

**Drug release profiles of SC@PDA-Gal**

2 mg SC@PDA-Gal was dissolved in 4 mL of different PBS buffer (pH 7.4 or pH 5.0), and stirred at 37°C continually. 500 μL of the solution was collected at indicated time points and then the same volume of solution was added. The absorbance was measured at different time points to calculate the amount of released SK by an UV-vis spectrometer. To study NIR-triggered release of drug, additional NIR light irradiation (808 nm, 1.5 W/cm^2^) was applied at indicated time points (1 h, 3 h, 5 h, 7 h, 9 h, 11 h) for 5 min. DLE (%) = (Actual mass of loaded drug / Theoretical mass of administered drug) × 100%, DEE (%) = (Mass of encapsulated drug / Theoretical total mass of administered drug) × 100%.

**Enzymatic Activity of SC@PDA-Gal**

The TMB/HRP colorimetric assay was used as a colorimetric probe to quantitatively evaluate enzyme activity - oxidized TMB has a characteristic absorption peak at 652 nm, and enzyme activity can be calculated based on the absorbance value at this wavelength.The experiment was conducted in a system containing 10 mM hydrogen peroxide (H_2_O_2_), with simultaneous addition of TMB/HRP and different concentrations of nanocarriers (SC@PDA-Gal). The enzyme activity was verified through changes in solution color and absorbance values.

**Studies of cellular uptake**

Cells were seeded in 48-well plates at a density of 2×10^4^ cells per well and cultured for 12 hours to allow adherence. For the galactose (Gal) competition group, galactose was added to the wells at a final concentration of 1 mM, followed by continued incubation for 12 hours .After washing the cells twice with PBS buffer , fresh medium containing SC@PDA-GR (at a final concentration of 20 μg/mL) was added to each well, and the cells were co-incubated with the medium for 6 hours .At the end of the co-incubation, the cells were stained with 4',6-diamidino-2-phenylindole (DAPI) at room temperature for 15 minutes . Finally, the distribution of RhB fluorescent signals of SC@PDA-GR in the cells was observed and recorded using a fluorescence microscope.

Cells were seeded in 6-well plates at a density of 3×10^6^ cells per well and cultured for 12 hours to allow adherence. For the galactose (Gal) competition group, galactose was added to the wells at a final concentration of 1 mM, followed by continued incubation for 12 hours. After washing the cells three times with PBS buffer, fresh medium containing SC@PDA-GR (20 μg/mL) was added to each well, and the cells were co-incubated with the medium for 6 hours. Finally, the cells were washed with PBS buffer and dispersed into a single-cell suspension, and the intracellular RhB fluorescence intensity was detected by flow cytometry.

**Cell culture and cytotoxicity assay**

C5WN1, HepG2, Huh7, SMMC-7721, and HEK293 cells were seeded in 96-well plates (1×104 cells per well) and allowed to attach for 12 h. The medium was then replaced with 100 μL of fresh culture medium containing PDA or SC@PDA-GR at various concentrations (equivalent PDA concentration: 0-96 μg mL^-1^), followed by incubation for 48 h. Cell viability was subsequently evaluated using a standard 3-(4,5-dimethylthiazol-2-yl)-2,5-diphenyltetrazolium bromide (MTT) assay, and the relative viability was calculated by normalizing to the untreated control.

For the photothermal treatment, after cell attachment the medium was replaced with 100 μL of fresh culture medium containing PDA or SC@PDA-GR (equivalent PDA concentration: 0-96 μg mL^-1^). After incubation for 24 h, the cells were irradiated with an 808 nm laser (1.5 W cm^-2^, 5 min). The cells were then further cultured for 24 h, and cell viability was determined by the MTT assay as described above.

In vitro cytotoxicity assay

Cells were seeded at 1 × 10^4^ cells/well in 96-well plates and were cultured for 12 h. The cells were then treated with Saline, PDA, PDA+NIR, SK, SK@PDA, SC@PDA, SC@PDA-Gal, SC@PDA-Gal NIR for 48 h. The medium was then replaced with MTT solution (20 μL, per well), which was cultured for 4 h at room temperature. Finally, the absorbance of each well was measured at 490 nm by a microplate reader. After 12 h of drug incubation, NIR group was irradiated with NIR light irradiation (808 nm, 1.5 W/cm^2^) for 10 min.

To quantify the synergistic therapeutic effect, the Bliss Independence Model was applied to analyze the interaction between targeted chemotherapy (SC@PDA-Gal) and phototherapy (PDA+NIR). *Iexp*=*I_A_*+*I_B_*-(*I_A_*×*I_B_*), ΔI=*Iobs-Iexp*, where *I_A_* is SC@PDA-Gal inhabit rate, *I_B_* is PDA +NIR inhabit rate, *Iobs* is SC@PDA-Gal+NIR inhabit rate, *Iexp* is theoretical expectation inhabition rate.

**Western blot assay**

Cells were seeded in 6-well plates at a density of 3 × 10^6^ cells per well and cultured for 12 hours. Subsequently, the cells were co-incubated with SK@PDA-GR and SC@PDA-GR for 24 hours. After that, the medium was removed, and the cells were washed with cold PBS buffer. A total of 100 μL of protein lysis buffer (RIPA: PMSF = 100:1) was added to each well, and the cells were lysed on ice for 15 minutes to obtain total protein. The protein was boiled at 100 °C for 10 minutes and then directly used for sample loading in Western Blot (WB) assay. The membrane was blocked in 5% non-fat milk at room temperature for 2 hours, followed by the addition of primary antibodies and incubation at 4 °C overnight. The membrane was washed 3 times with TBST, and then incubated with horseradish peroxidase-conjugated secondary antibodies at room temperature for 2 hours. After another 3 washes with TBST, the blot membrane was imaged using a Gel Doc™ XR + Gel Documentation System.

**The detection experiments for ATP and lactic acid contents**

The detection of ATP and Lactatce was performed in accordance with the kit instructions.

**Xenograft Tumor Models**

The C5WN1 cell xenograft tumor models were established according to a previous method [5]. All animal experimental protocols were reviewed and approved by the Animal Committee of Jiangnan University (JN. No20241230b0720630). 5-weeks old male BALB/c nude mice were injected with 2 × 10^6^ C5WN1 cells (0.1 mL, PBS/Matrigel, v/v 1:1) in the right hind dorsum. The length and width of tumor were measured by a vernier caliper every day to calculate volume. according to the formula below, tumor volume = 0.5 × (tumor width)^2^ × (tumor length). When the tumor volume was 50 mm3, the mice were treated with different formulations.


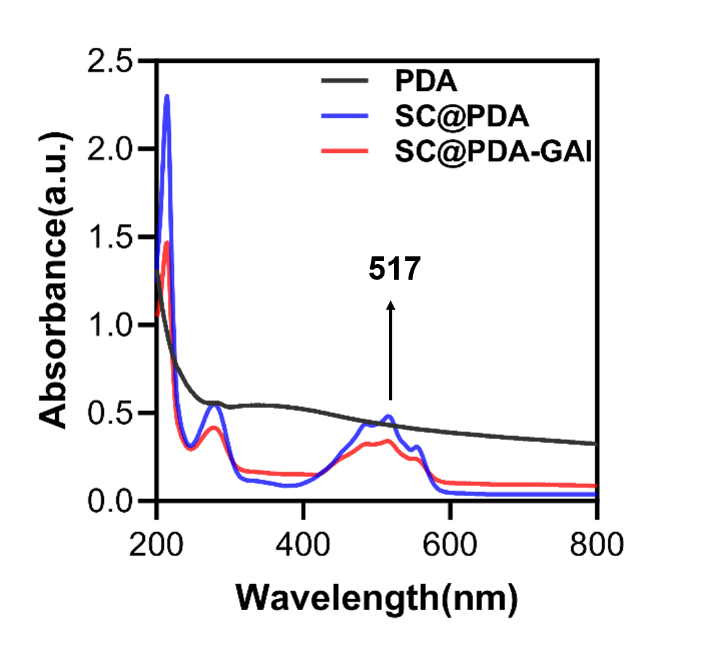


**Figure S1.** UV−vis absorption spectra of PDA, SC@PDA and SC@PDA-Gal.


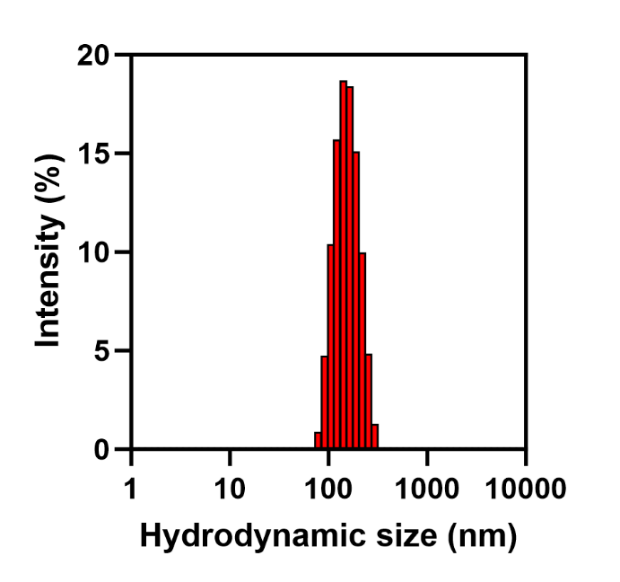


**Figure S2.** Hydrodynamic size distribution of PDA.


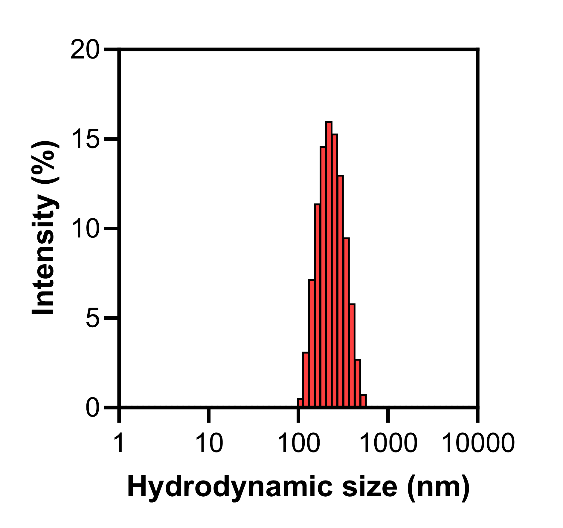


**Figure S3.** Zeta Hydrodynamic size distribution of SC@PDA-Gal.

**Table S1. DLE (Drug Loading Efficiency) and DEE (Drug Encapsulation Efficiency) of SK and CAT.**

| (PDA/SK)/CAT  （m/m）/m | DLE (%) | | DEE (%) | |
| --- | --- | --- | --- | --- |
|  | SK | CAT | SK | CAT |
| (1:0.5):2 | 20.69±0.02 | 7.34±0.06 | 62.09±0.08 | 22.01±0.02 |
| (1:1):2 | 19.39±0.17 | 6.42±0.14 | 65.52±1.16 | 25.68±0.55 |
| (1:2):2 | 26.02±0.12 | 4.50±0.12 | 51.89±0.09 | 18.00±0.48 |


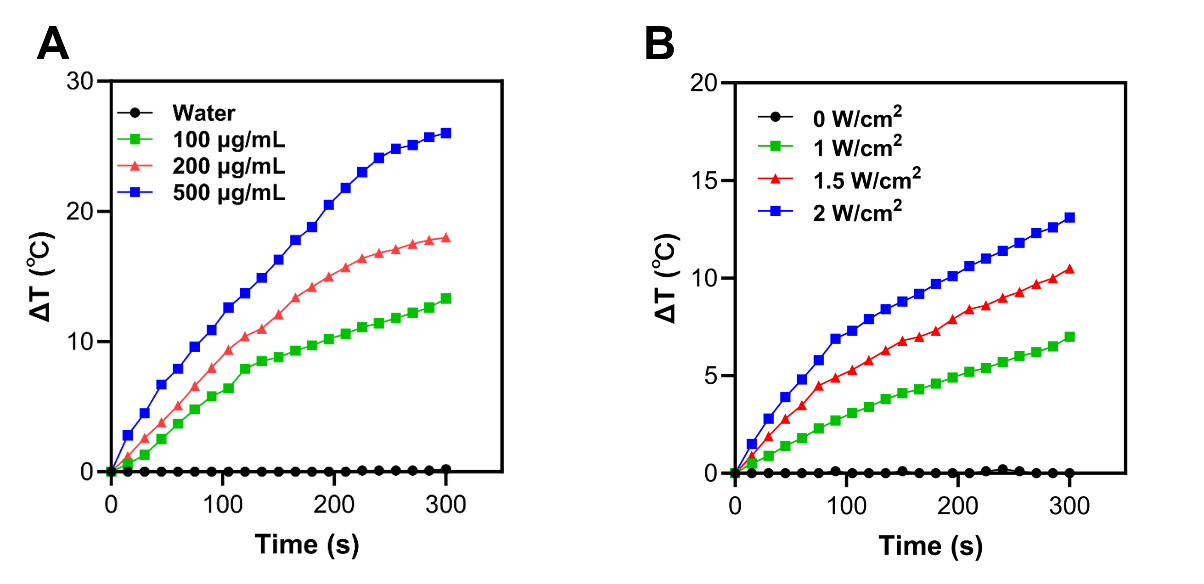


**Figure S4.** (A) Temperature variation curves of different concentration of SC@PDA-Gal exposed to the 808 nm NIR irradiation (5 min, 1.5W/cm2). (B) Temperature variation curves of same concentration of SC@PDA-Gal exposed to the 808 nm NIR irradiation (100 μg/mL, 5 min).


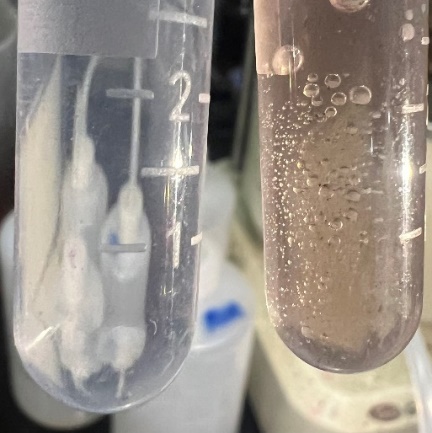


**Figure S5.** Changes in oxygen production by PBS, SC@PDA-Gal in 10 mM H_2_O_2_.

**
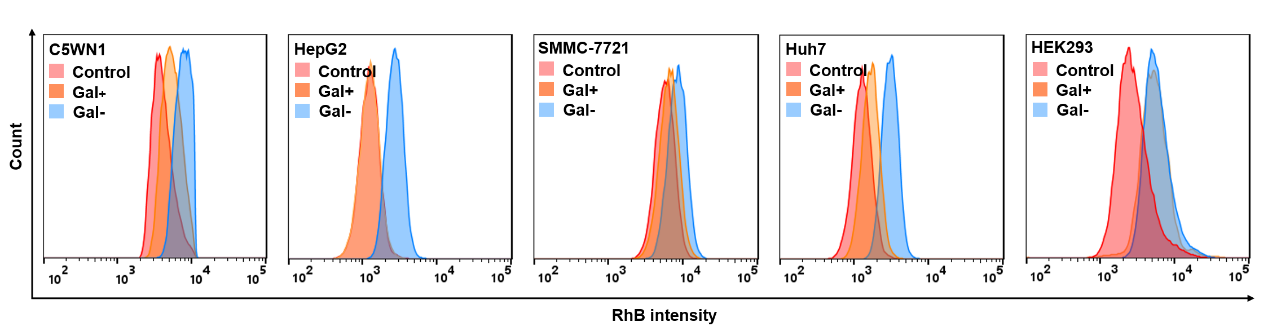
**

**Figure S6.** Flow cytometry analysis results of C5WN1, HepG2, SMMC-7721, Huh7 and HEK293 cells co-incubated with SC@PDA-Gal-RhB in the presence or absence of 1 mM galactose (Gal) competition.

**
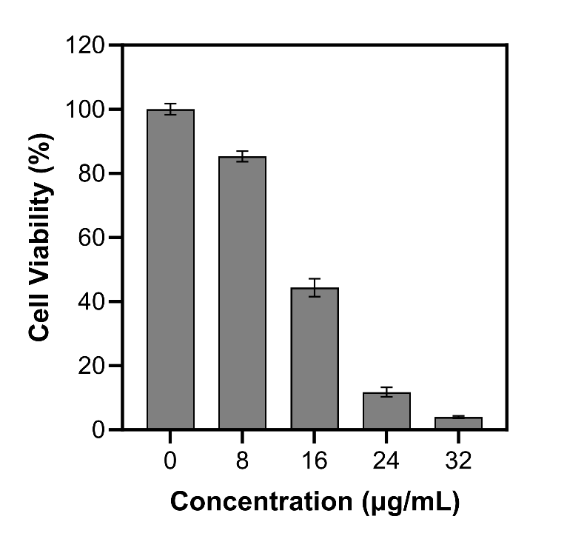
**

**Figure S7.** Cell viability of C5WN1 cells incubated with SC@PDA-Gal at various concentrations for 48 h.


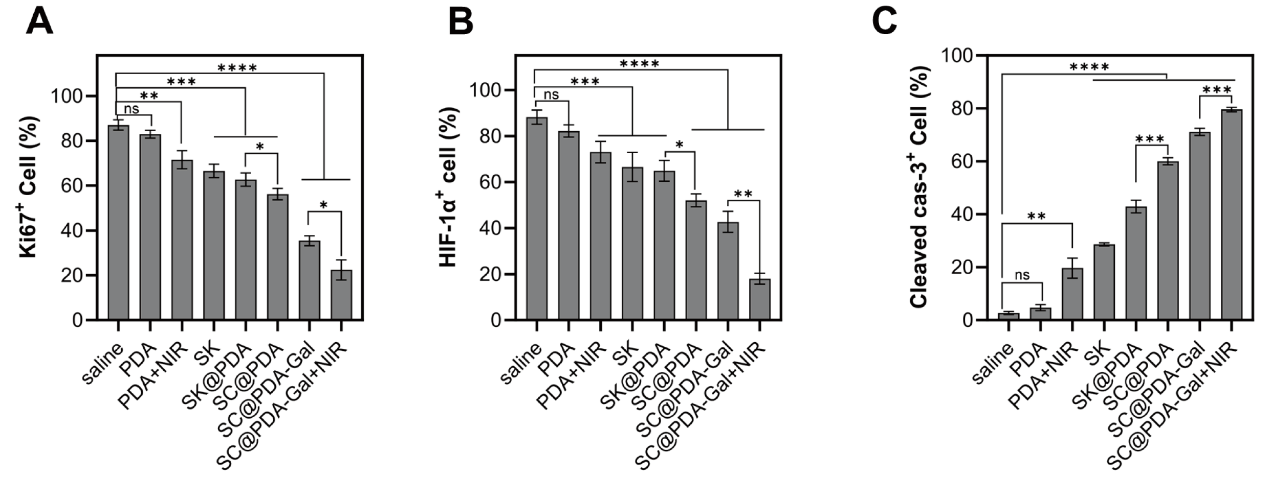


**Figure S8.** Quantitative analysis of the positive staining areas for (A) Ki67, (B) HIF-1α and (C) Cleaved caspase-3 based on the IHC images in Fig. 5E. **p < 0.01, ***p < 0.001, ****p < 0.0001. Mean ± SD (n = 3).


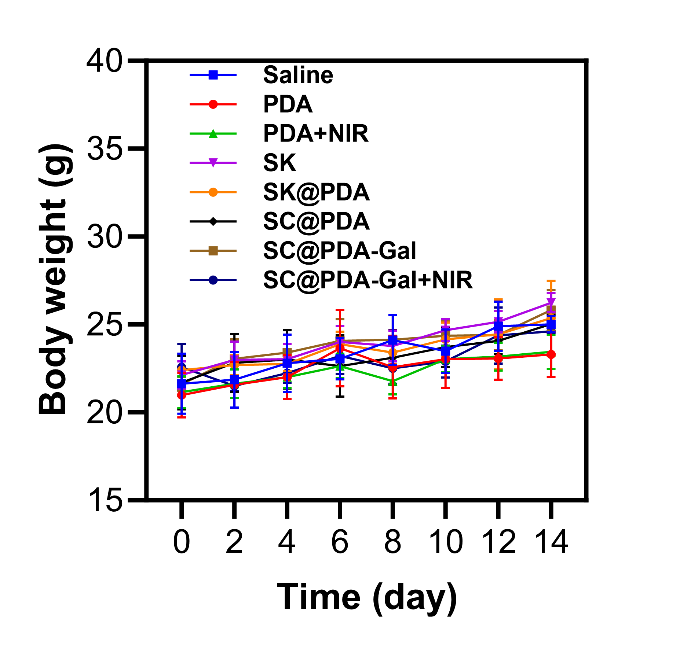


**Figure S9**. Average body weight changes of tumor-bearing mice during the 14-day treatment.


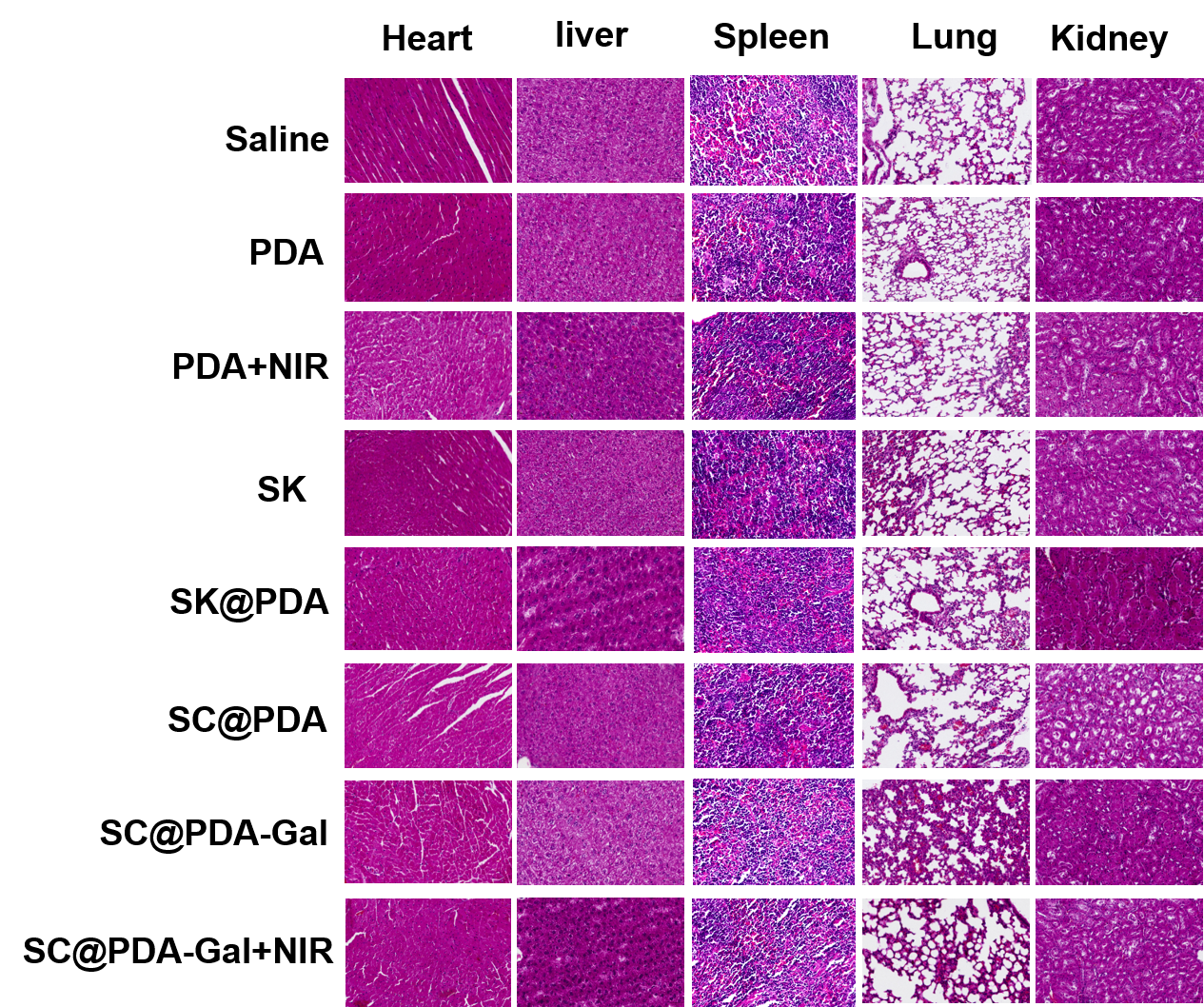


**Figure S10.** H&E staining of major organs in the tumor-bearing mice after the 14-day treatment. Scale bar: 50 µm.
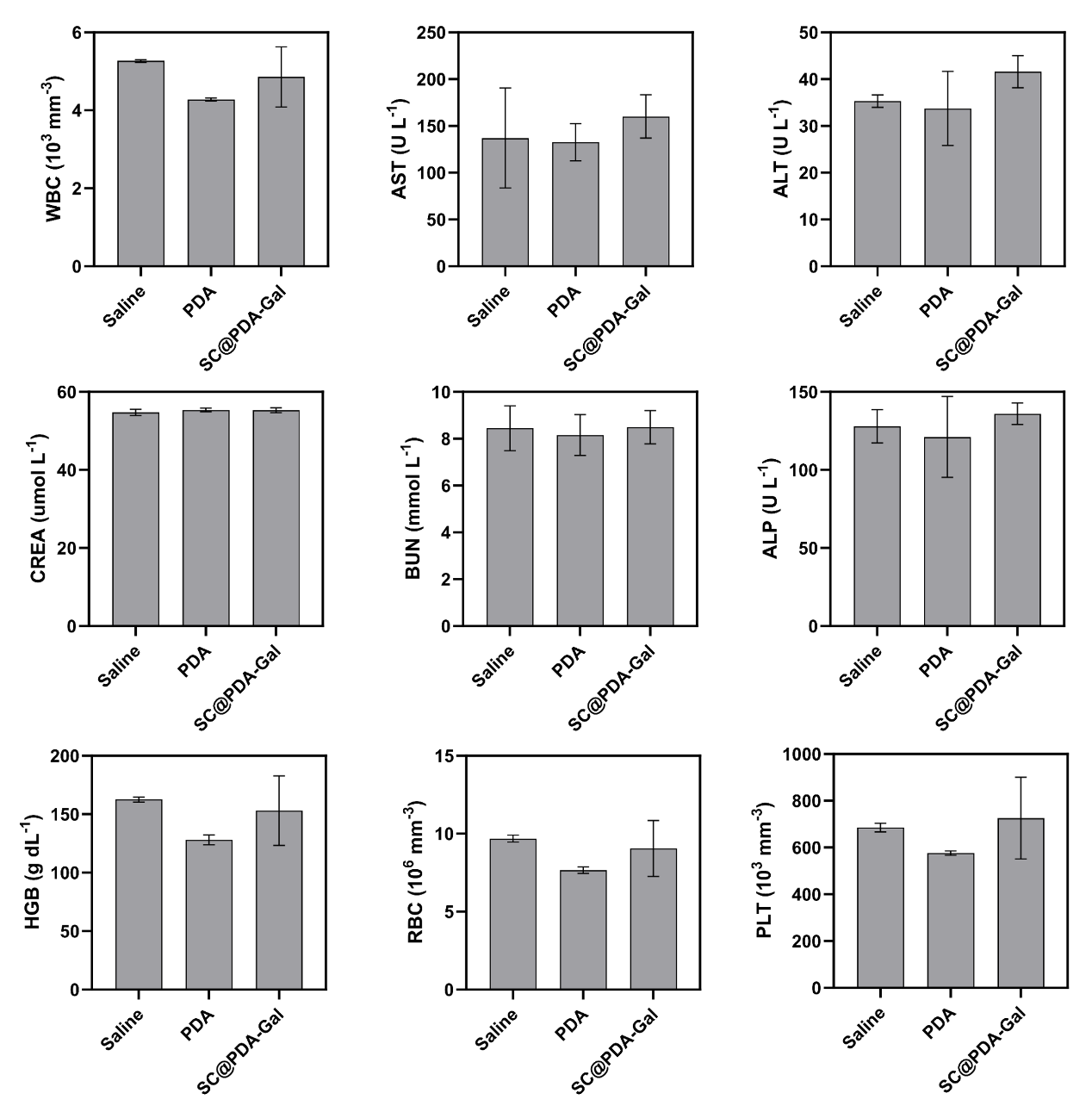


**Figure S11**. The changes of blood routine and biochemical indexes of tumor-bearing mice in different groups after the 14-day treatment.
